# Assessing the risk zones of Chagas' disease in Chile, in a world marked by global climatic change

**DOI:** 10.1590/0074-02760170172

**Published:** 2018-01

**Authors:** Valentina Tapia-Garay, Daniela P Figueroa, Ana Maldonado, Daniel Frías-Laserre, Christian R Gonzalez, Alonso Parra, Lucia Canals, Werner Apt, Sergio Alvarado, Dante Cáceres, Mauricio Canals

**Affiliations:** 1 Universidad de Chile, Facultad de Medicina, Escuela de Salud Pública, Programa de Salud Ambiental, Santiago, Chile; 2 Universidad de Chile, Facultad de Ciencias Veterinarias y Pecuarias, Departamento de Ciencias Biológicas Animales, Santiago, Chile; 3 Universidad Metropolitana de Ciencias de la Educación, Departamento de Entomología, Santiago, Chile; 4 Ministerio de Salud, Control de Vectores, Santiago, Chile; 5 Universidad de Chile, Facultad de Medicina, Laboratorio de Parasitología, Santiago, Chile; 6 Universidad de Chile, Facultad de Medicina, Departamento de Medicina, Santiago, Chile

**Keywords:** Chagas' disease, Triatoma infestans, distribution, climate change, Chile

## Abstract

**BACKGROUND:**

Vector transmission of *Trypanosoma cruzi* appears to be interrupted in Chile; however, data show increasing incidence of Chagas' disease, raising concerns that there may be a reemerging problem.

**OBJECTIVE:**

To estimate the actual risk in a changing world it is necessary to consider the historical vector distribution and correlate this distribution with the presence of cases and climate change.

**METHODS:**

Potential distribution models of *Triatoma infestans* and Chagas disease were performed using Maxent, a machine-learning method.

**FINDINGS:**

Climate change appears to play a major role in the reemergence of Chagas' disease and *T. infestans* in Chile. The distribution of both *T. infestans* and Chagas' disease correlated with maximum temperature, and the precipitation during the driest month. The overlap of Chagas' disease and *T. infestans* distribution areas was high. The distribution of *T. infestans*, under two global change scenarios, showed a minimal reduction tendency in suitable areas.

**MAIN CONCLUSION:**

The impact of temperature and precipitation on the distribution of *T. infestans*, as shown by the models, indicates the need for aggressive control efforts; the current control measures, including *T. infestans* control campaigns, should be maintained with the same intensity as they have at present, avoiding sylvatic foci, intrusions, and recolonisation of human dwellings.

Chagas' disease is one of the most prevalent, yet neglected, diseases in the Americas and an emerging disease in other locations throughout America and Europe. It has been compared to the early stages of the HIV/SIDA pandemic. Annual incidence varies from 28,000 to 56,000 individuals, with 10,000 to 14,000 annual deaths (Hotez et al. 2012), affecting 6-11 million individuals (Cucunubá et al. 2016), but with 65 to 100 million at risk (MINSAL 2014, 2016). In Chile, the endemic area is located between the Arica-Parinacota (18°30'S) and Libertador Bernardo O'Higgins (34°36'S) regions, where approximately 900,000 individuals remain at risk (MINSAL 2014).

Chagas' disease is a protozoan infection caused by *Trypanosoma cruzi*, transmitted in Chile by the kissing bug vectors *Triatoma infestans*, *Mepraia spinolai*, *Mepraia gajardoi* and *Mepraia parapatrica* (Hemiptera, Reduviidae, Triatominae); *T. infestans* is the domiciliary vector (Apt & Reyes 1986a, b, Canals et al. 1992, Botto-Mahan et al. 2005, Frías-Laserre et al. 2017) and the main species responsible for the prevalence of this disease in Chile and in the Americas (Canals et al. 1993). The protozoan can also be transmitted via transfusion and congenital, oral, and accidental routes; however, these are of minor importance in Chile (Nóbrega et al. 2009, MINSAL 2014).

The last national health report (ENS) indicated a population prevalence of 0.7%, with 1.5% and 0.6% in rural and urban zones, respectively (MINSAL 2009-2010, 2016), and a very low domiciliary infestation rate (MINSAL 2016). The data in these reports are in contrast with those reported in the 1980s and 1990s. For example, between 1937-1980 the prevalence was 16.7% in rural endemic zones with a maximum of 43.6% in the Coquimbo region (Schenone et al. 1980). This did not vary in the 1982-1985 period (Schenone et al. 1985), while between 1982-1989, a prevalence of 1.9% was reported in urban zones (Schenone & Rojas 1989). Previously reported domiciliary infestation data indicated rates between 26.8% and 33.2% between the Arica and Libertador Bernardo O'Higgins regions (Schenone et al. 1980, MINSAL 2016).

Since 1999 in Chile, 1997 in Uruguay, 2006 in Brazil, and recently (since 2016) in Paraguay, vector transmission by *T. infestans* has been interrupted as a consequence of efficient eradication campaigns (MINSAL 2014, Rojas de Arias 2016). This interruption could have changed the dynamics of Chagas' disease from vector to congenital transmission, with consequences in the reproductive number (R0), prevalence, incidence and trypano-triatomine indices (Massad 2008, Rojas de Arias 2016, Canals et al. 2017a, b). This creates a false impression that Chagas' disease is not a problem in these countries, which has consequences on efforts made toward prevention and control. Thus, this disease is neglected (Hotez et al. 2012, Rojas de Arias 2016).

There are some data in Chile that show increasing incidence (MINSAL 2016) and reports of sylvatic foci of the main vector, *T. infestans* (Bacigalupo et al. 2010, Canals et al. 2017b). In this scenario, it is difficult to obtain an accurate impression of the real risk of Chagas' disease in this country.

Moreover, the world is experiencing social, political and climatic changes that may have consequences on the distribution of vectors and the prevalence of infectious diseases. A paradigmatic example is the variation in the distribution of *Aedes aegypti* and, consequently, on the distribution of dengue between 1930 and the present. This mosquito initially inhabited a large region throughout the Americas and the Caribbean; its distribution was reduced to some Caribbean zones in 1970. However, currently, the distribution is similar to, or greater than, the original distribution (Gubler 2008), including northern Chile, with an explosive increment in dengue in the last few years. For this reason, it is necessary to consider the historical distribution of the vectors (ecological maps) and correlate these distributions with cases (incidence-based maps) to estimate the risk of vector-borne diseases in a changing world.

In this article, we assess the historical distribution of *T. infestans*, the main and domestic vector of Chagas' disease in Chile, comparing current distribution with the original distribution where the dominant transmission form was vectors. We then explore the changes in the distribution of vectors under two climate change scenarios.

## MATERIALS AND METHODS

*Vector occurrence data* - Data on the occurrence of *T. infestans* in Chile were obtained from the National Museum of Natural History, Entomology Institute of Metropolitan University of Educational Sciences, Health Ministry of Chile, Public Health Institute and literature reports, covering the time-period from 1943, onwards. Duplicate and erroneous records were excluded. Records in oversampled locations were also excluded based on subsampling among very close pairs of points, to reduce sampling bias (Peterson et al. 2008). Points of occurrence were separated by at least 1 km. Data were filtered following the criteria: (1) the information must be accurately geo-referenced (2) the data must include the name of the zoologist who determined the species, to avoid taxonomic problems. A total of 222 occurrences were considered and after filtering we obtained a total of 110 points of occurrence.

*Chagas' disease occurrence data* - We obtained occurrence data for Chagas' disease from the archives of the Parasitology Laboratory of the Medicine Faculty of the University of Chile. Records between 1939-1965 were considered because, during this period, the transmission was mainly by *T. infestans* (Schenone et al. 1980). The data were organised in an Excel file, geo-referencing the location (if the information was available). In Chile, there are regional administrative divisions; each region is divided into communes. When the exact address was not recorded, the geometric centre of the residence commune was geo-referenced. Geo-referencing was done using geographic coordinates Datum WGS 84 (World Geodetic System). This method considers some spatial uncertainty (*sensu* Peterson & Samy 2016). To estimate this, we used the average radius over all communes S_u_ = Σr_i_/n with r_i_ the radius of a circumference, with equal area of each commune “i”. This was a broad estimate of the spatial uncertainty. Also, similarly to vector occurrence data, error and oversampled locations were avoided. A total of 3395 cases were considered, and after filtering, 219 cases were included in the model.

*Climatic and environmental data* - The dataset of environmental variables was composed of proxy bioclimatic variables obtained from the Worldclim database (http://www.worldclim.org/), with two spatial resolutions: 3 arcsec (≈1 km^2^); this dataset included a total of 19 bioclimatic variables that summarised temperature and precipitation data. Seven variables were selected from this set, considering their relationship to the distribution of Triatomines: B1 = mean annual temperature; B4 = temperature seasonality (standard deviation*100); B5 = maximum temperature in the warmest month; B6 = minimum temperature in the coldest month; B12 = annual precipitation; B14 = precipitation during the driest month and B15 = precipitation seasonality (coefficient of variation). These variables were chosen considering that *T. infestans* populations are affected by temperature and precipitation, including two variables that consider loads (B1 and B12) and information regarding the deviations of these variables (B4 and B15). We also included extreme temperature variables (B5 and B6) because extreme temperatures exert known effects on development, dispersion, and mortality of *T. infestans* (Canals et al. 1992, 2016).

*Global change scenarios* - The same variables used previously, but for 2070 (average between 2061-2080), were obtained from the Worldclim database for global climate model (GCM) in two global change scenarios: optimistic (RCP: 2.6) and pessimistic (RCP: 8.5), performed by the Instituto Nacional de Pesquisas Espaciais (INPE) of Brazil with a resolution of 2.5 arc-min (≈5 km^2^).

*Analyses* - Spatial distribution models were constructed for occurrences of the vector and Chagas' disease cases using Maxent, a machine-learning method that assesses the distribution probability of a case or species by estimating the maximum entropy probability distribution; it is a proven method with very good results. We used bootstrap subsampling with 30 replicated and random seeds, and the mean of replicates. The model was smoothed to avoid over-parametrisation (Peterson et al. 2008). The Maxent output was converted to binary maps using an error rate of 10%.

Considering that: (1) The southern boundary of the distribution of *T. infestans* in Chile is not well known and was only established in a single, dated study; (2) Humans aid this insect's dispersal capacity, and there are reports of colonies of these insects in trains (Faundez 2016); (3) The aim of this study was to compare the distribution of patients with Chagas's disease; there are permanent ministerial reports of cases of Chagas' disease in latitudes further to the South (Bio-Bio, Araucania and De los Ríos Regions: 36°-40°33'S). The models were not calibrated based on hypotheses of accessible areas “M” for these vectors (Barve et al. 2011). Thus, for comparative purposes, we extended the model to the whole Chilean national territory. To study the model goodness-of-fit, we used the area under the curve (AUC) in the receiver operating characteristic (ROC) analysis.

To compare the current distribution model of *T. infestans* to the distribution model for cases of Chagas' disease and the distribution model of *T. infestans*, under the optimistic and pessimistic global change scenarios, a reclassification function was used in DIVA-GIS: 1 for a probability of occurrence > 0.2 and 0 otherwise. This procedure allowed estimation of the suitable areas (km^2^), and the areas of superposition.

## RESULTS

The distribution models for Chagas' disease and for *T. infestans* showed a good fit (AUC = 0.957 ± 0.005 and AUC = 0.954 ± 0.010, respectively). For both models the maximum temperature in the warmest month and precipitation in the driest month contributed considerably to the distribution. For the Chagas' disease map, annual precipitation, temperature seasonality, and average temperature were also relevant (Table). A broad estimation of the spatial uncertainty was S_u_ = 17.7 ± 18.0 km.

**TABLE:** Contribution of the bioclimatic variables to the models of Chagas' disease and *Triatoma infestans* distribution

Variable	Chagas disease distribution	*T. infestans* distribution
B14 = precipitation during the driest month	27.6	29.7
B5 = maximum temperature in the warmest month	27.0	51.5
B12 = annual precipitation	10.8	-
B4 = temperature seasonality	8.4	-
B1 = mean annual temperature	8.0	-
Total	81.8	81.2

**Fig. 1:**
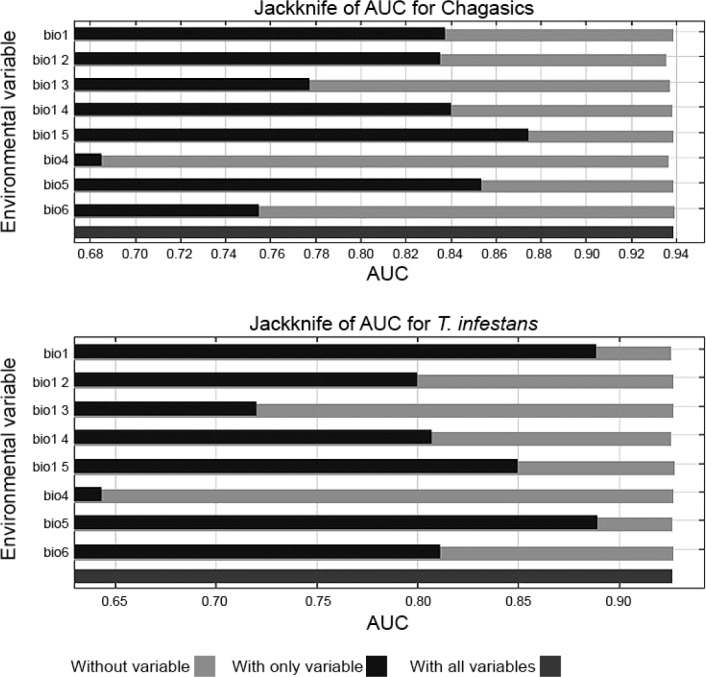
jackknife of the area under receiver operating characteristic (ROC) curves (AUC) for Chagas' disease cases and for *Triatoma infestans* distribution models.

**Fig. 2:**
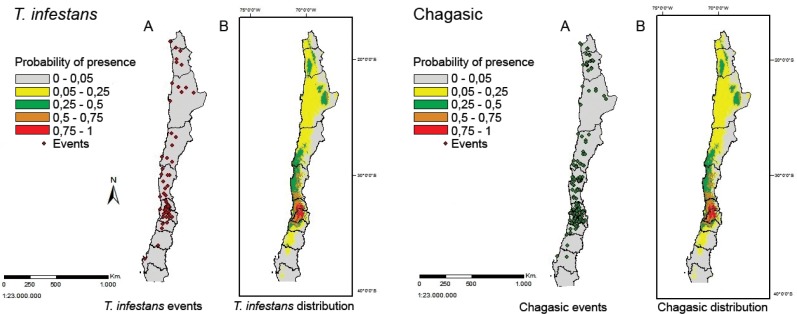
distribution maps for *Triatoma infestans* (left) and Chagas' disease cases (right: A). Points represents the empirical presence data (B) map of the potential distribution. From north to south, the lines show the limits of the Chilean regions: the first eight are: Arica y Parinacota, Tarapaca, Antofagasta, Atacama, Coquimbo, Valparaiso, Metropolitana, Bernardo O'Higgins, and Maule.

**Fig. 3:**
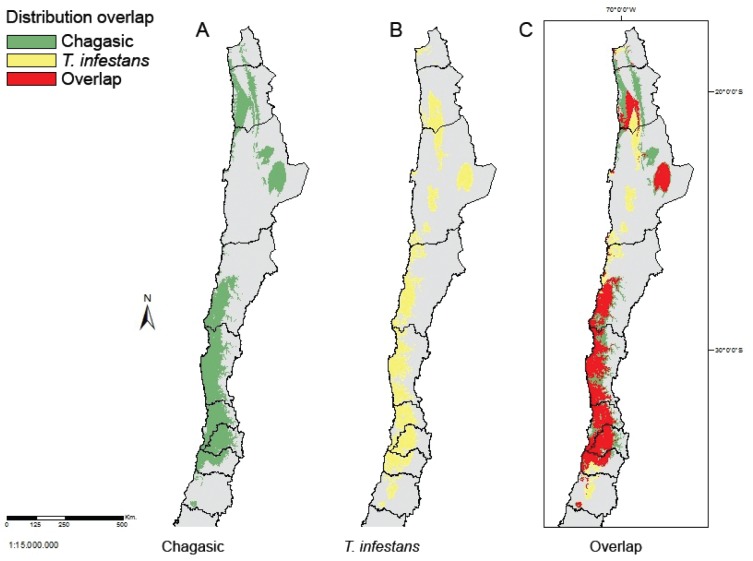
map of the distribution overlap of Chagas' disease cases and *Triatoma infestans* with a threshold value of 0.2 for presence. From north to south, the lines show the limits of the Chilean regions: the first eight are: Arica y Parinacota, Tarapaca, Antofagasta, Atacama, Coquimbo, Valparaiso, Metropolitana, Bernardo O'Higgins, and Maule.

The jackknife method showed that, for Chagas' disease, precipitation seasonality and maximum temperature during the warmest month were the best predictor variables. For *T. infestans* distribution, these were maximum temperature in the warmest month and the mean annual temperature (Fig. 1), accounting for more than 0.85 in the AUC. The potential distributions of Chagas' disease and *T. infestans* were similar (Fig. 2). The area occupied by Chagas' disease was 109,034 km^2^ and the *T. infestans* distribution was 90,829 km^2^ with an overlap of 67796 km^2^, representing 51.33% of the total area (Fig. 3).

**Fig. 4:**
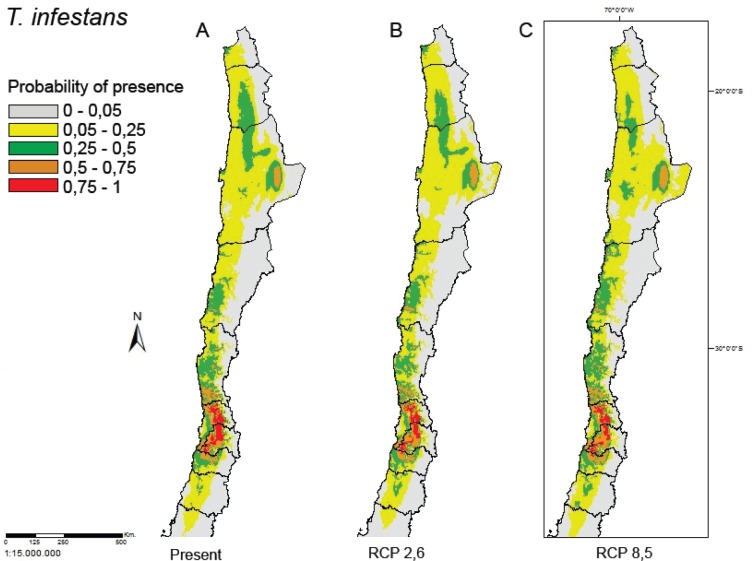
map of the estimation of the potential distribution of *Triatoma infestans* under two scenarios of climactic change: (A) actual scenario (B) optimistic RCP: 2.6, and (C) pessimistic RCP: 8.5.

The distribution of *T. infestans* under the two climatic change scenarios studied showed low variation with a minimal reduction tendency in suitable areas (Fig. 4). In the benign scenario, the suitable area was 99.33% of the current area, and in the pessimistic scenario it was 93.64% of the current area, with overlap percentages of 92.01% and 91.67%, respectively.

## DISCUSSION

The distribution of cases of Chagas' disease covers an area slightly larger than the distribution area of *T. infestans*. This is an expected result because of internal population migratory movements. The distribution of *T. infestans* is consistent with that usually reported for this species with a southern limit in the O' Higgins region. For Chagas' disease cases and for *T. infestans*, the zones with high presence probability were Antofagasta, Coquimbo, Valparaiso, and the Metropolitan region (Santiago), in agreement with frequently-reported data (Schenone et al. 1980, Apt & Reyes 1986a, b, Schenone & Rojas 1989) and prevailing in Mediterranean zones in the interior valleys, which feature arid and semi-arid weather. For example, recent data show that the highest incidence was reported in Coquimbo, which along with Antofagasta, Coquimbo, and the Metropolitan region encompass approximately two-thirds of the notified cases (MINSAL 2014, 2016). Although the Ministry of health reported a prevalence of 0.1 to 0.5/100000 habitants in latitudes that are farther south (Bio-Bio, Araucania and De los Ríos Regions: 36°S-40°33'S) where *T. infestans* is not reported, our data and suitable distribution maps do not support this report, suggesting that those cases likely represent immigrants from endemic zones.

Our results agree with the potential distribution maps reported for *T. infestans*, based on surveys (Hernández et al. 2013). Hernández et al. (2013) reported that the presence probability distribution increases towards the North (Coquimbo Region), tending to cover the central-coastal region, and avoiding areas of the Andes Range. Also, they proposed a tendency towards lower probabilities in the South, near the Pacific coast. Our results are consistent with observed tendencies for avoiding the Andes Range; although the Coquimbo, Valparaiso and Metropolitan regions had the most suitable areas, the presence probability was more concentrated in the central zone. This has two possible explanations. The first is that Hernández et al. (2013) worked with surveys exploring the presence of the two insect vectors (*T. infestans* and *M. spinolai*), and we only were interested in *T. infestans*, the domestic vector. The second is that there may be a sample bias in our occurrence data, since the central zone of Chile is always the most studied due to the good climatic conditions and, in contrast to the amplitude of the northern desert, prevents good sampling of the area.

The overlap between *T. infestans* and the Chagas' disease cases was 51.33% considering areas with a presence probability greater than 0.2 (threshold) as “suitable”. This estimation is sensitive to the threshold value used; the lower the threshold, the higher the overload percentage. Comparing the distribution maps of Chagas' disease cases and *T. infestans* with empirically reported occurrences (Fig. 2), the threshold value 0.2 appears to be reasonable because the occurrences were distributed mainly in zones with presence probabilities greater than 0.2 (green, orange and red areas in Fig. 2). There was an area of complete overlap (51.33%), areas where *T. infestans* is probable but there are no cases of Chagas' disease (17.44%), and areas where Chagas' disease cases are probable but the areas are not suitable for *T. infestans* (31.22%). The suitable areas for *T. infestans*, but not for Chagas' disease, are zones with probable underreporting, with low human density, or with low bug population density. For example, the Sierra Gorda and Calama communes where the reports of domiciliary infestation were fewer than 1% during a period where there were few control efforts (Burchard et al. 1984). The zones with Chagas' disease cases, but without *T. infestans*, are probably zones with subsampling of bugs. The zones of least coincidence were Tarapacá and Antofagasta, particularly the latter which is a desert zone of low population density with undersampling of bugs and underreporting of Chagas cases.

The climate change predictions in Chile include an increase of temperature over the entire nation, with a gradient of higher to lower temperatures, from north to south and from the Andes to the Pacific Ocean. This increase in temperature is less than expected considering the predicted rates of mean global warming. During the period of 2011-2030, the temperature increase would be about 0.5°C in the south zone and 1.5°C for the north and the Chilean Altiplano. During the period of 2031-2050, the warming pattern would be maintained; however, the increments would be greater (up to 2°C). It is expected that the greatest warming would be at high altitudes in the Andes Range of northern Chile. In the coast, warming will be modest (0.5 to 1°C) and may increase up to 5°C in the Andes (Garreaud 2011). A decrease in precipitation, between 5% and 15%, is expected during the period of 2011-2030 between the basins of the Copiapo and the Aysen Rivers (27°S-45°S). This decrease in precipitation will intensify during the 2031-2050 period (Garreaud 2011). Our results suggest that these changes will only produce a small effect on the distribution of *T. infestans*, with a slight reduction in suitable areas. This is consistent with the decrease in suitable areas proposed for the species *M. spinolai* in the same area, and is in contrast with the high impact on the distribution of *M. gajardoi*, a species with a small distribution in the coast of northern Chile (Garrido 2017). Under the assumption of niche conservatism, the latter species would suffer disappearance of its habitat, while *M. gajardoi*, a species with distributions similar to *T. infestans* and with similar preferred environmental conditions, would decrease its distribution area in the interior valleys while increasing its distribution on the coast (Garrido 2017), like *T. infestans*. Since *T. infestans* is a species residing in arid and semi-arid habitats, its distribution area would not be affected significantly, maintaining the transmission risk of Chagas' disease in this zone. Thus, suitable areas for the development of sylvatic foci and human dwelling intrusions in Chile will be maintained under climate change. The campaigns for control of *T. infestans* should be maintained with the same intensity as they exhibit at present, avoiding sylvatic foci, intrusions, and re-colonisation of human dwellings.

## References

[B1] Apt W, Reyes H (1986a). Aspectos epidemiológicos de la enfermedad de Chagas en Chile. I:
Distribución geográfica, índices de infección en vectores y en
humanos. Parasitol Día.

[B2] Apt W, Reyes H (1986b). Aspectos epidemiológicos de la enfermedad de Chagas en Chile. II:
Infección en animales, algunas características especiales del problema, el
control. Parasitol Día.

[B3] Bacigalupo A, Torres-Pérez F, Segovia V, García A, Correa JP, Moreno L (2010). Sylvatic foci of the Chagas disease vector *Triatoma
infestans* in Chile: description of a new focus and challenges
for control programs. Mem Inst Oswaldo Cruz.

[B4] Barve N, Barve V, Jiménez-Valverde A, Lira-Noriega A, Maher SP, Peterson AT (2011). The crucial role of the accessible area in ecological niche
modeling and species distribution modeling. Ecol Model.

[B5] Botto-Mahan C, Ortiz S, Rozas M, Cattan PE, Solari A (2005). DNA evidence of *Trypanosoma cruzi* in the Chilean
wild vector *Mepraia spinolai* (Hemiptera:
Reduviidae). Mem Inst Oswaldo Cruz.

[B6] Burchard L, Cornejo J, Cruz M, Contreras MC, Vargas F, Villarroel F (1984). Epidemiología de la enfermedad de Chagas en Chile. Sectores
rurales. Infestación triatominea domiciliaria e infección por
*Trypanosoma cruzi* del vector y en mamíferos silvestres
de la II región, Chile. Bol Chil Parasitol.

[B7] Canals M, Alvarado S, Cáceres D, Cattan PE (2016). Twenty years of monitoring of mortality and fecundity of
*Triatoma infestans* in the laboratory. Parasitol Latinoam.

[B8] Canals M, Caceres D, Alvarado S, Canals A, Cattan PE (2017a). Modeling the Chagas disease: from the vectorial to congenital
transmission. Biosystems.

[B9] Canals M, Cattan PE, Ehrenfeld M, Torres P (1992). Poblaciones experimentales de *T. infestans*:
efectos de condiciones ambientales variables. Parasitol Día.

[B10] Canals M, Cattan PE, Ehrenfeld M (1993). Algunas estimaciones numéricas de la importancia epidemiológica
de los vectores de la enfermedad de Chagas en Chile. Parasitol Día.

[B11] Canals M, González C, Canals L, Canals A, Cáceres D, Alvarado S (2017b). Que dicen los números de la evolución temporal de la enfermedad
de Chagas?. Rev Chil Infectol.

[B12] Cucunubá ZM, Okuwoga O, Basáñez MG, Nouvellet P (2016). Increased mortality attributed to Chagas disease: a systematic
review and meta-analysis. Parasit Vectors.

[B13] Faundez E (2016). Sobre los registros aislados de *Triatoma
infestans* (Klug, 1834) (Heteróptera, Reduviidae) en el sur de
Chile. Arq Entomol.

[B14] Frías-Laserre D, González CR, Reyes C, de Carvalho DB, Oliveira J, Canals M (2017). Wing polymorphism and *Trypanosoma cruzi*
infection in wild, peridomestic and domestic collections of *Mepraia
spinolai* (Hemiptera: Reduviidae) from Chile. J Med Entomol.

[B15] Garreaud R (2011). Cambio climático: bases físicas e impacto en
Chile. Tierra Adentro.

[B16] Garrido R (2017). Impacto del cambio climático en la distribución geográfica de dos
vectores silvestres de la enfermedad de Chagas en Chile, *Mepraia
spinolai* y *Mepraia gajardoi* (Hemiptera:
Reduviidae).

[B17] Gubler DJ (2008). The global threat of emergent/reemergent
vector-borne diseases. Vector-Borne Diseases: understanding the environmental, human health,
and ecological connections; Institute of Medicine.

[B18] Hernández J, Núñez I, Bacigalupo A, Cattan PE (2013). Modeling the spatial distribution of Chagas disease vectors using
environmental variables and people's knowledge. Int J Health Geogr.

[B19] Hotez PJ, Dumonteil E, Woc-Colburn L, Serpa JA, Bezek S, Edwards MS (2012). Chagas Disease: “The New HIV/AIDS of the
Americas”. PLoS Negl Trop Dis.

[B20] Massad E (2008). The elimination of Chagas disease from Brazil. Epidemiol Infect.

[B21] MINSAL - Ministerio de Salud de Chile (2009-2010). Encuesta Nacional de Salud - Chile 2009-2010.

[B22] MINSAL - Ministerio de Salud de Chile (2016). Informe Programa Nacional Integral de Enfermedad de Chagas, Julio
2016.

[B23] MINSAL - Ministerio de Salud de Chile (2014). Norma general técnica. Control y prevención nacional de la enfermedad de
Chagas.

[B24] Nóbrega AA, García MH, Tatto E, Obara MT, Costa E, Sobel J (2009). Oral transmission of Chagas disease by consumption of acai palm
fruit, Brazil. Emerg Infect Dis.

[B25] Peterson AT, Samy AM (2016). Geographic potential of disease caused by Ebola and Marburg
viruses in Africa. Acta Trop.

[B26] Peterson AT, Soberon J, Pearson RG, Anderson RP, Martínez-Meyer E, Nakamura M (2008). Ecological niches and geographic distributions.

[B27] Rojas de Arias A (2016). La certificación del corte de transmisión vectorial del
*Trypanosoma cruzi*, agente etiológico de la enfermedad
de Chagas. Mem Inst Investig Cienc Salud.

[B28] Schenone H, Christensen HA, de Vásquez AM (1985). Fuentes de alimentación de triatomas domésticos y su implicancia
epidemiológica en relación a la enfermedad de Chagas en áreas rurales de
siete regiones de Chile. Bol Chil Parasitol.

[B29] Schenone H, Rojas A (1989). Algunos datos y observaciones pragmáticas en relación a la
epidemiologia de la enfermedad de Chagas. Bol Chil Parasitol.

[B30] Schenone H, Villarroel F, Rojas A, Alfaro E (1980). Factores biológicos y ecológicos en la epidemiología de la
enfermedad de Chagas en Chile. Bol Chil Parasitol.

